# Effects of Dietary Zearalenone on Oxidative Stress, Cell Apoptosis, and Tight Junction in the Intestine of Juvenile Grass Carp (*Ctenopharyngodon idella*)

**DOI:** 10.3390/toxins11060333

**Published:** 2019-06-12

**Authors:** Ya-Li Wang, Xiao-Qiu Zhou, Wei-Dan Jiang, Pei Wu, Yang Liu, Jun Jiang, Shang-Wen Wang, Sheng-Yao Kuang, Ling Tang, Lin Feng

**Affiliations:** 1 Animal Nutrition Institute, Sichuan Agricultural University, Chengdu 611130, China; wangyali@stu.sicau.edu.cn (Y.-L.W.); zhouxq@sicau.edu.cn (X.-Q.Z.); WDJiang@sicau.edu.cn (W.-D.J.); wupei0911@sicau.edu.cn (P.W.); 11081@sicau.edu.cn (Y.L.); jjun@sicau.edu.cn (J.J.); 2 Fish Nutrition and safety Production University Key Laboratory of Sichuan Province, Sichuan Agricultural University, Chengdu 611130, China; 3 Key Laboratory for Animal Disease-Resistance Nutrition, Chengdu 611130, China; 4 Key Laboratory of Animal Disease-Resistant Nutrition, Ministry of Education, Chengdu 611130, China; 5 Key Laboratory of Animal Disease-Resistant Nutrition and Feed, Ministry of Agriculture and Rural Affairs, Chengdu 611130, China; 6 Tongwei Research Institute, Tongwei Co., Ltd., Chengdu 600438, China; Wangsw@tongwei.com; 7 Animal Nutrition Institute, Sichuan Academy of Animal Science, Sichuan Animtech Feed. Co., Ltd., Chengdu 610066, China; shengyao.kuang@gmail.com (S.-Y.K.); lingtang.fish@gmail.com (L.T.)

**Keywords:** zearalenone, intestine, oxidative injury, apoptosis, tight junctions

## Abstract

Zearalenone (ZEA) is a prevalent mycotoxin with high toxicity in animals. In order to study its effect on juvenile grass carp (*Ctenopharyngodon idella*), six diets supplemented with different levels of ZEA (0, 535, 1041, 1548, 2002, and 2507 μg/kg diet) for 10 weeks were studied to assess its toxicity on intestinal structural integrity and potential mechanisms of action. Our report firstly proved that ZEA led to growth retardation and body deformity, and impaired the intestinal structural integrity of juvenile grass carp, as revealed by the following findings: (1) ZEA accumulated in the intestine and caused histopathological lesions; (2) ZEA resulted in oxidative injury, apoptosis, and breached tight junctions in the fish intestine, which were probably associated with Nuclear factor-erythroid 2-related factor 2 (Nrf2), p38 mitogen activated protein kinases (p38MAPK), and myosin light chain kinase (MLCK) signaling pathways, respectively. ZEA had no influence on the antioxidant gene levels of *Kelch-like ECH associating protein 1* (*Keap1)b* (rather than *Keap1a*), *glutathione-S-transferase* (*GST)P1*, *GSTP2* (not in the distal intestine (DI)), tight junctions *occludin*, *claudin-c* (not in the proximal intestine (PI)), or *claudin-3c* (not in the mid intestine (MI) or DI).

## 1. Introduction

Mycotoxins are defined as natural secondary metabolites found in a number of crops, including aflatoxin, zearalenone (ZEA), deoxynivalenol (DON), ochratoxin A (OTA), and T-2 toxin [1,2,3]. So far, studies of the toxic effects of mycotoxins on fish have primarily been focused on growth inhibition and liver injury [4,5]. However, the intestine is the first physiological barrier in the case of feed contamination, for example, by dietary mycotoxin in animals [6]. Reports of the toxic influence of mycotoxins on fish intestinal structure are scarce, and the following restrictions still remain: (1) Available studies are lacking in systematicity and depth. Except for the previous study on DON from our lab [7], most other studies have only investigated the apparent morphology [8]; (2) The toxic effect on the intestine is different among various mycotoxins. For example, in rainbow trout (*Oncorhynchus mykiss*), T-2 toxin-induced intestinal lesions (such as intestinal hemorrhage) occurred [9], whereas DON had no effect on the intestinal structure [10]. Thus, it is essential to systematically study the influence of other mycotoxins on fish intestinal structure and investigate their mechanisms of toxicity in depth.

ZEA is one of the most prevalent mycotoxins, and has high toxicity in animals [11,12]. The prevalence of ZEA in common plant feedstuffs of soybean meal, cotton seed meal, and wheat products has been found to be up to 85.54, 120.89, and 145.30 µg/kg, respectively, whereas the average concentration of ZEA in the same feedstuffs has been found to 18.90, 14.12, and 18.64 µg/kg, respectively [13,14]. A maximal ZEA concentration of 511 µg/kg feed was found in commercial fish feed [15]. There have been many studies of the effect of ZEA on mammals, however, these mainly focused on reproductive toxicity [16,17]. Meanwhile, only a few studies have reported the influence of ZEA on fish. These mainly focused on developmental toxicity (such as body malformation in the fathead minnow (*Pimephales promelas*)) [18] and the impairment of reproduction in zebrafish (*Danio rerio*) [19]. Additionally, some studies investigated growth performance, serum parameters, and liver and kidney toxicity [5,20,21]. As mentioned above, the intestine is the first physiological barrier in the case of feed contamination by mycotoxin, which also means the intestine is the first organ targeted by dietary mycotoxin. However, only three studies have investigated the effect of ZEA on fish intestines. Two studies reported that ZEA could accumulate in the intestine of rainbow trout [22,23], while another study demonstrated that ZEA did not result in lipid peroxidation in carp (*Cyprinus carpio L*.) intestines [24]. Hence, it is necessary to carry out systematic and in-depth research on the influence of ZEA on the fish intestinal structure. A study reported that ZEA increased the levels of RAW264.7 cellular reactive oxygen species (ROS) in mice, which could lead to cell death [25]. Excessive cell death has been found to lead to excessive damage to the structure of ovaries in pigs [26]. The destructive effect of toxic and harmful substances on the intestinal structure of rats has been shown to be closely associated with the destruction of cellular structure (antioxidant ability and cell apoptosis) and intercellular structure (tight junction proteins (TJs)) [27], which could be modulated by Nuclear factor-erythroid 2-related factor 2 (Nrf2), p38 mitogen activated protein kinases (p38MAPK), and myosin light chain kinase (MLCK) signaling pathways in fish intestines [28], respectively. It was reported that the presence of ZEA in the head kidney cells of common carp increased the production of nitric oxide [20], which may cause the activation of Nrf2 in human vascular cells [29]. Therefore, it is necessary to study the toxic effect of ZEA on intestinal structural integrity in fish, as well as its possible mechanism of toxicity.

The aim of the current study was to explore the effect of ZEA exposure on antioxidant ability, apoptosis, and the tight junctions and relevant signaling pathways in fish intestinal tissue. Additionally, we assessed the dose of no observed adverse effect levels (NOAEL) for ZEA on juvenile grass carp (*Ctenopharyngodon idella*).

## 2. Results

### 2.1. Growth Performance and Body Malformation of Fish

Table 1 presents the influences of ZEA on the growth indicators of juvenile fish. The ZEA ≥ 1041 μg/kg diet depressed the percentage of weight gain (PWG), specific growth rate (SGR), final body weight (FBW), feed intake (FI), feed efficiency (FE), intestinal length (IL), intestinal length index (ILI), intestinal weight (IW), and intestinal somatic index (ISI). Additionally, the ZEA concentrations in fish intestinal tissues all increased when ZEA levels were raised.

**Figure 1:**
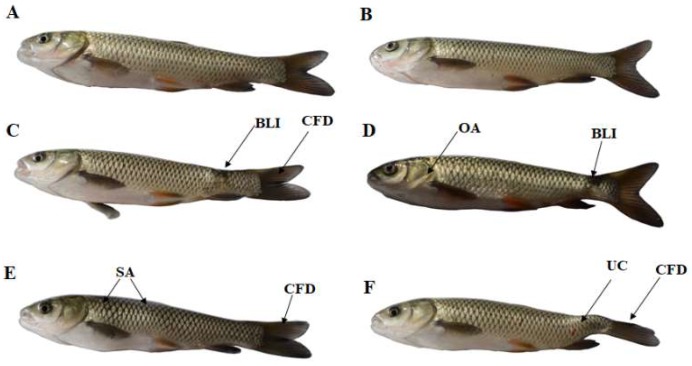
The body malformation of juvenile grass carp (*Ctenopharyngodon idella*) fed diets containing graded levels of ZEA for 10 weeks. (**A**): control; (**B**): 535 μg/kg diet; (**C**): 1041 μg/kg; (**D**): 1548 μg/kg diet; (**E**): 2002 μg/kg diet; (**F**): 2507 μg/kg diet. Abbreviations for deformities analysis of juvenile grass carp: BLI, body line irregularities; CFD, caudal fin deformity; OA, operculum abnormality; SA, skeletal anomalies; UC, upward curvature of the tail.

Figure 1 presents the influences of ZEA on body deformity in juvenile fish. In the 0 and 535 μg ZEA/kg groups, no body malformation was observed. However, body line irregularities (BLI) and caudal fin deformity (CFD) were observed in the 1041 μg/kg group, BLI and operculum abnormality (OA) were observed in the 1548 μg/kg group, skeletal anomalies (SA) and CFD were observed in the 2002 μg/kg group, and upward curvature of the tail (UC) and CFD were observed in the 2507 μg/kg group. 

### 2.2. Inflammation and Histopathological Changes

**Figure 2:**
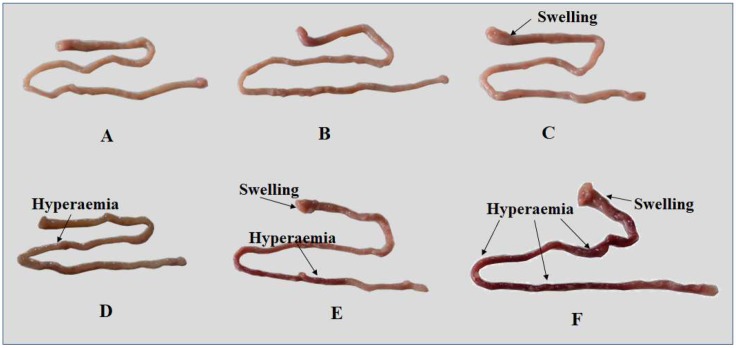
The intestinal lesions of juvenile grass carp (*Ctenopharyngodon idella*) fed diets containing graded levels of ZEA for 10 weeks. (**A**): control; (**B**): 535 μg/kg diet; (**C**): 1041 μg/kg diet; (**D**): 1548 μg/kg diet; (**E**): 2002 μg/kg diet; (**F**): 2507 μg/kg diet.

**Figure 3:**
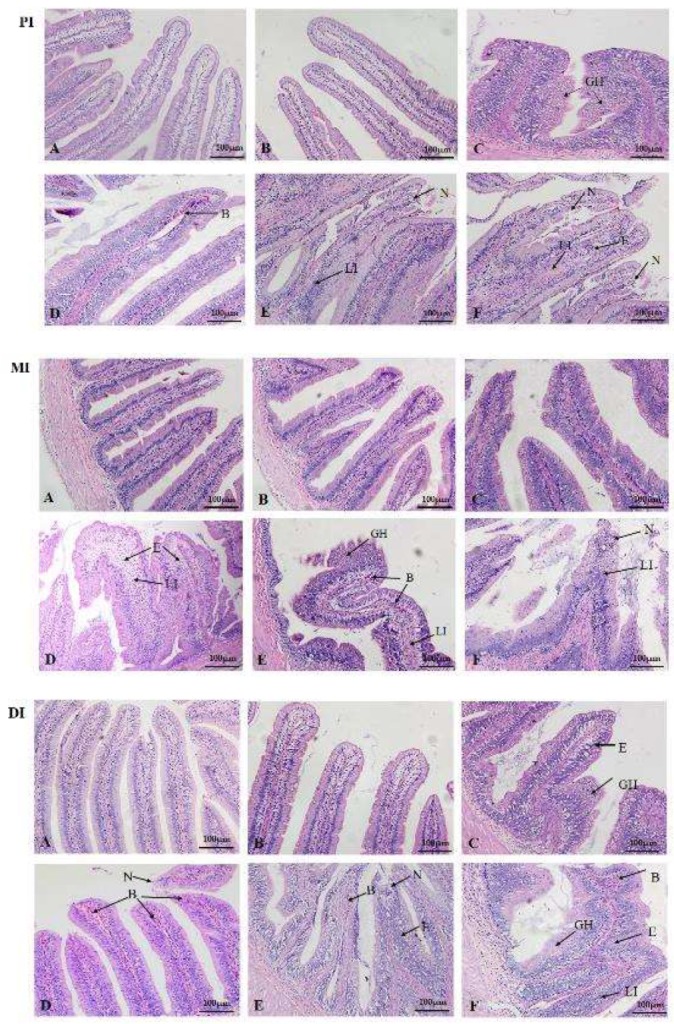
The histology of the PI, MI, and DI in juvenile grass carp fed diets containing graded levels of ZEA (μg/kg diet). (**A**) control; (**B**) 535 μg/kg diet; (**C**) 1041 μg/kg diet; (**D**) 1548 μg/kg diet; (**E**) 2002 μg/kg diet; (**F**) 2507 μg/kg diet. In each panel, N: necrosis; LI: leucocyte infiltration; GH: goblet cell hyperplasia; B: blood capillary hyperemia; E: edema in the lamina propria. H&E staining, magnification ×200.

Compared to the control group, we observed intestinal swelling in the 1041 μg/kg diet group, hyperemia in the 1548 μg/kg diet group, and swelling and hyperemia in the 2002 and 2507 μg ZEA/kg diet groups, respectively (Figure 2). The histological results show that ZEA resulted in edema in the lamina propria, leucocyte infiltration, necrosis of epithelial cells, blood capillary hyperemia, and goblet cell hyperplasia in the proximal intestine (PI), mid intestine (MI), and distal intestine (DI) of fish (Figure 3). In the PI, goblet cell hyperplasia was observed in the 1041 μg/kg diet group, blood capillary hyperemia was observed in the 1548 μg ZEA/kg diet group, leucocyte infiltration and epithelial cell necrosis were observed in the ZEA of the 2002 μg/kg diet group, and edema in the lamina propria, leucocyte infiltration, and epithelial cell necrosis were observed in the 2507 μg/kg diet group. In the MI, the 1548 μg/kg ZEA diet was found to induce leucocyte infiltration and edema in the lamina propria. Additionally, goblet cell hyperplasia, blood capillary hyperemia, and leucocyte infiltration were found in the 2002 μg/kg diet group. Furthermore, leucocyte infiltration and epithelial cell necrosis were observed in the 2507 μg/kg diet group. In the DI, goblet cell hyperplasia and edema in the lamina propria were observed in the ZEA of the 1041 μg/kg diet group. Blood capillary hyperemia and epithelial cell necrosis were observed in the ZEA of the 1548 μg/kg diet group. Blood capillary hyperemia, epithelial cell necrosis, and edema in the lamina propria were observed in the 2002 μg/kg diet group. Meanwhile, leucocyte infiltration, blood capillary hyperemia, goblet cell hyperplasia, and edema in the lamina propria were found in the DI of the 2507 μg/kg diet group.

### 2.3. Oxidative Injury, Antioxidant-Relevant Parameters, and Gene Expressions of Relevant Signalling Molecules 

As displayed in Table 2, the production of ROS, protein oxidation (PC), and lipid peroxidation (MDA) in the intestine increased with increasing ZEA dose. Relative to the control group, the production of ROS in the PI was markedly higher with increasing ZEA levels in the 1548 μg/kg diet group (*p* < 0.05), while the productions of MDA and PC in the PI were markedly higher with increasing ZEA levels in the 1041 μg/kg diet group (*p* < 0.05). The productions of ROS and PC in the MI and DI were markedly higher with increasing ZEA levels in the 1041 μg/kg diet group (*p* < 0.05), while the production of MDA in the MI and DI was markedly higher with increasing ZEA levels in the 1548 μg/kg diet group (*p* < 0.05). The activities of the anti-superoxide anion (ASA), anti-hydroxyl radical (AHR), copper/zinc superoxide dismutase (CuZnSOD), manganese superoxide dismutase (MnSOD), catalase (CAT), glutathione peroxidase (GPx), glutathione-S-transferase (GST) and glutathione reductase (GR), and glutathione (GSH) production in the intestinal tissue of fish were markedly down-regulated by ZEA compared to the control group (*p* < 0.05). In the PI, compared to the control group, a significant decrease was observed in CuZnSOD, GST, and GR activities in the 1041 μg ZEA/kg diet (*p* < 0.05); CAT activity was markedly reduced in the 1548 μg/kg diet group (*p* < 0.05); MnSOD and GPx activities were markedly reduced in the 2002 μg/kg diet group (*p* < 0.05); and ASA and AHR activities were markedly reduced in the 2507 μg ZEA/kg diet group (*p* < 0.05). In the MI, a significant decrease in GR activity was observed in the 535 μg/kg diet group (*p* < 0.05); the activities of ASA, CuZnSOD, and GST were markedly reduced in the 1041 μg/kg diet group (*P* < 0.05); MnSOD and GPx activities were markedly reduced in the 1548 μg/kg diet group (*p* < 0.05); AHR activity was markedly reduced in the 2002 μg/kg diet group (*p* < 0.05); CAT activity was markedly reduced in the 2507 μg ZEA/kg diet group (*p* < 0.05), compared with the control group. In the DI, compared with the control group, the activities of GPx, GST, and GR were markedly reduced in the 1041 μg/kg diet group (*p* < 0.05), whereas the activities of ASA, AHR, CuZnSOD, MnSOD, and CAT were markedly reduced in the 1041 μg/kg diet group (*p* < 0.05). In the 2507 μg ZEA/kg diet, GSH production was markedly reduced in the three intestinal segments (*p* < 0.05).

**Figure 4:**
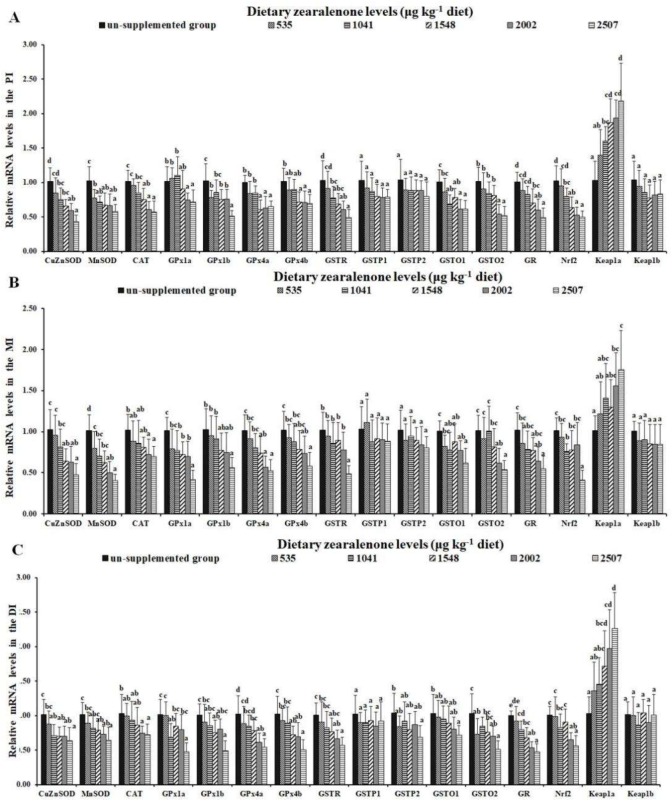
Relative mRNA levels of *CuZnSOD, MnSOD, CAT, GPx1a, GPx1b, GPx4a, GPx4b, glutathione-S-transferase (GST)P1, GSTP2, GSTO1, GSTO2, GR, Nuclear factor-erythroid 2-related factor 2 (Nrf2), Kelch-like ECH-associated protein 1 (Keap1)a* and *Keap1b* in the PI (**A**), MI (**B**), and DI (**C**) of juvenile grass carp fed diets containing graded levels of zearalenone. Data represent means of six fish in each group, error bars indicate SD. Values having different letters are significantly different (*p* < 0.05).

As presented in Figure 4A, the gene expressions of *MnSOD* and *GPx1b* in the PI were markedly decreased in the 535 μg/kg diet group (*p* < 0.05); the mRNA levels of *CuZnSOD, glutathione-S-transferase (GST)R, GSTO1,* and *Nrf2* were markedly reduced in the 1041 μg/kg diet group (*p* < 0.05); mRNA levels of *CAT, GPx4a, GPx4b,* and *GR* were markedly decreased in the 1548 μg/kg diet group (*p* < 0.05); and mRNA levels of *GPx1a* and *GSTO2* were markedly reduced in the 2002 μg/kg diet group (*p* < 0.05), compared to the control group. As presented in Figure 4B, compared to the control group, mRNA levels of *MnSOD* and *GPx1a* were significantly decreased in the 535 and 1041 μg/kg diet groups (*p* < 0.05), respectively; mRNA levels of *CuZnSOD* and *GPx4a* were markedly reduced in the 1548 μg/kg diet group (*p* < 0.05); mRNA levels of *CAT, GPx4b, GSTO2,* and *GR* were markedly reduced in the 2002 μg/kg diet group (*p* < 0.05); and mRNA levels of *GPx1b, GSTR, GSTO1,* and *Nrf2* were markedly reduced in the 2507 μg ZEA/kg diet group (*p* < 0.05) in the MI. As presented in Figure 4C, compared with the control group, a significant decrease in the mRNA level of *CuZnSOD* was observed in the 1041 μg/kg diet group (*p* < 0.05); mRNA levels of *MnSOD, GPx1b, GPx4a,* and *GPx4b* were markedly reduced in the 1548 μg/kg diet group (*p* < 0.05); mRNA levels of *CAT* and *GSTR* were significantly decreased in the 2002 μg/kg diet group (*p* < 0.05); mRNA levels of *GPx1a, GSTP2, GSTO1,* and *GSTO2* were markedly reduced in the 2507 μg ZEA/kg diet group (*p* < 0.05) in the DI. Additionally, the gene expressions of *Kelch-like ECH-associated protein 1* (*Keap1)a* in the PI, MI, and DI of fish were markedly increased in the 1041, 2002, and 1548 μg/kg diet groups (*p* < 0.05), respectively. It is interesting that ZEA had no influence on the gene expressions of *GSTP1* and *Keap1b* in the three intestinal segments, or on the gene expression of *GSTP2* in the PI and MI of fish (*p* > 0.05).

**Figure 5:**
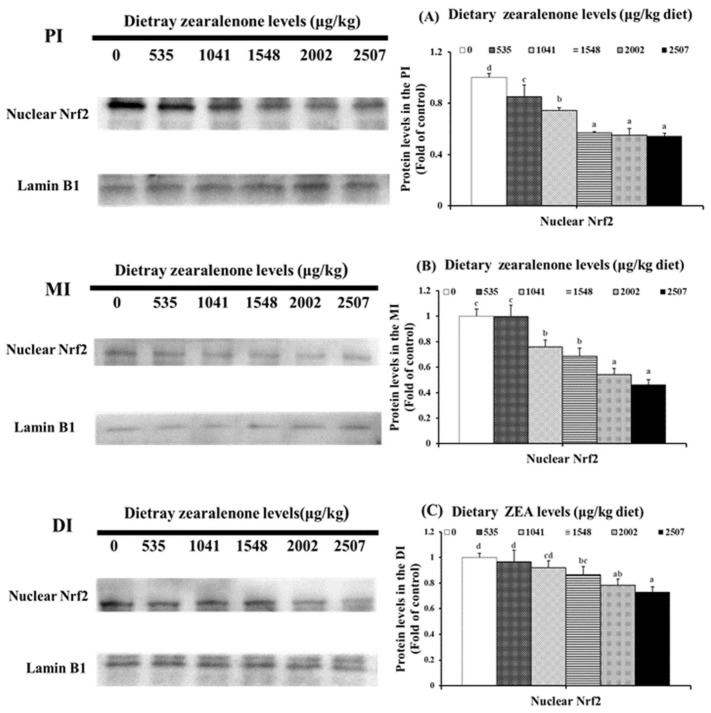
Western blot analysis of nuclear Nrf2 in the PI (**A**), MI (**B**), and DI (**C**) of juvenile grass carp (*Ctenopharyngodon idella*) fed diets containing graded levels of ZEA for 10 weeks. Values having different letters are significantly different (*p* < 0.05).

As displayed in Figure 5, compared with the control group, a marked decrease in nuclear Nrf2 protein expressions was observed in the PI, MI, and DI of fish in the 535, 1041, and 1548 μg/kg diet groups (*p* < 0.05), respectively.

### 2.4. DNA Fragmentation, Apoptosis-Related Indicators, and Signalling Molecules 

**Figure 6:**
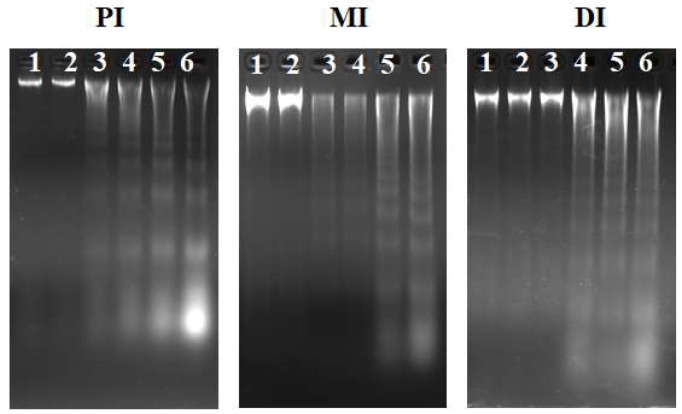
Effects of different dietary ZEA levels on DNA fragmentation in the PI, MI, and DI of juvenile grass carp using agarose gel electrophoresis. Lane 1: control group. Lane 2–Lane 6: levels of dietary ZEA were 535, 1041, 1548, 2002, and 2507 μg/kg, respectively. This experiment was repeated three times with similar results achieved.

**Figure 7:**
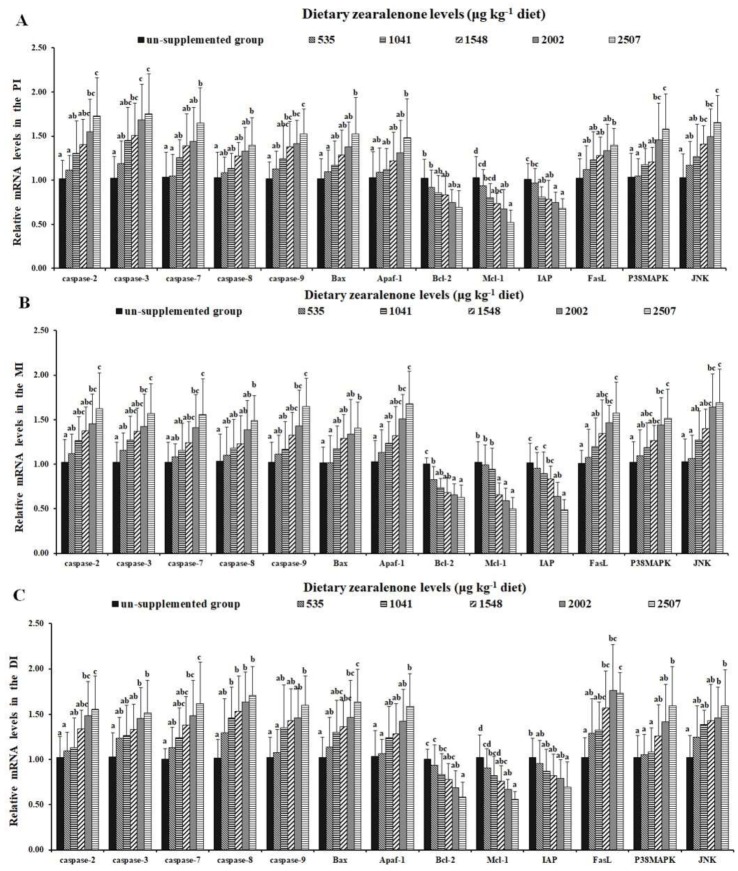
Relative mRNA levels of *cysteinyl aspartate specific proteinase (caspase)-2, caspase-3, caspase-7, caspase-8, caspase-9, Bax, Apaf-1, Bcl-2, Mcl-1, IAP, FasL*, and *p38MAPK* in the PI (**A**), MI (**B**), and DI (**C**) of juvenile grass carp fed diets containing graded levels of zearalenone. Data represent means of six fish in each group, error bars indicate SD. Values having different letters are significantly different (*p* < 0.05).

Figure 6 presents the influences of ZEA on DNA laddering in the PI, MI, and DI of fish. In this study, DNA fragmentation was found in the intestine. As presented in Figure 7A, it was found that the gene expressions of *cysteinyl aspartate specific proteinase (caspase)-3, caspase-9,* and *c-Jun N-terminal kinases (JNK)* were markedly up-regulated in the 1548 μg/kg diet group (*p* < 0.05); mRNA levels of *caspase-2* and *p38MAPK* were markedly up-regulated in the 2002 μg/kg diet group (*p* < 0.05); and mRNA levels of *caspase-7, caspase-8, B-cell lymphoma protein 2 associated X protein (Bax)*, *apoptotic protese activating factor-1 (Apaf-1),* and *Fas ligand* (*FasL)* were markedly reduced in the 2507 μg ZEA/kg diet group (*p* < 0.05) in the PI. As presented in Figure 7B, mRNA levels of *caspase-2, caspase-3*, *caspase-7, caspase-9, Apaf-1, p38MAPK, FasL,* and *JNK* were markedly up-regulated in the 2002 μg/kg diet group (*p* < 0.05); whereas mRNA levels of *caspase-8* and *Bax* were markedly reduced in the 2507 μg ZEA/kg diet group (*p* < 0.05) in the MI. As presented in Figure 7C, mRNA levels of *caspase-8* and *p38MAPK* were markedly up-regulated in the 1041 and 1548 μg/kg diet groups (*p* < 0.05), respectively; mRNA levels of *caspase-2, caspase-3*, *caspase-7*, *Bax, and JNK* were markedly up-regulated in the 2002 μg/kg diet group (*p* < 0.05); and mRNA levels of *caspase-9, Apaf-1,* and *FasL* were markedly reduced in the 2507 μg ZEA/kg diet group (*p* < 0.05) in the DI. The gene expressions of *B-cell lymphoma-2* (*Bcl-2)*, *inhibitor of apoptosis protein* (*IAP),* and *myeloid cell leukemia-1 (Mcl-1)* in the PI were markedly decreased in the 2507, 1041, and 1548 μg/kg diet groups (*p* < 0.05), respectively; the gene expressions of *Bcl-2*, *IAP,* and *Mcl-1* in the MI were markedly decreased in the 535, 2002, and 1541 μg/kg diet groups (*p* < 0.05), respectively; and the gene expressions of *Bcl-2*, *IAP,* and *Mcl-1* in the DI were markedly decreased in the 2002, 2507, and 1548 μg/kg diet groups (*p* < 0.05), respectively.

### 2.5. Tight Junctions and Relevant Signalling Molecules

**Figure 8:**
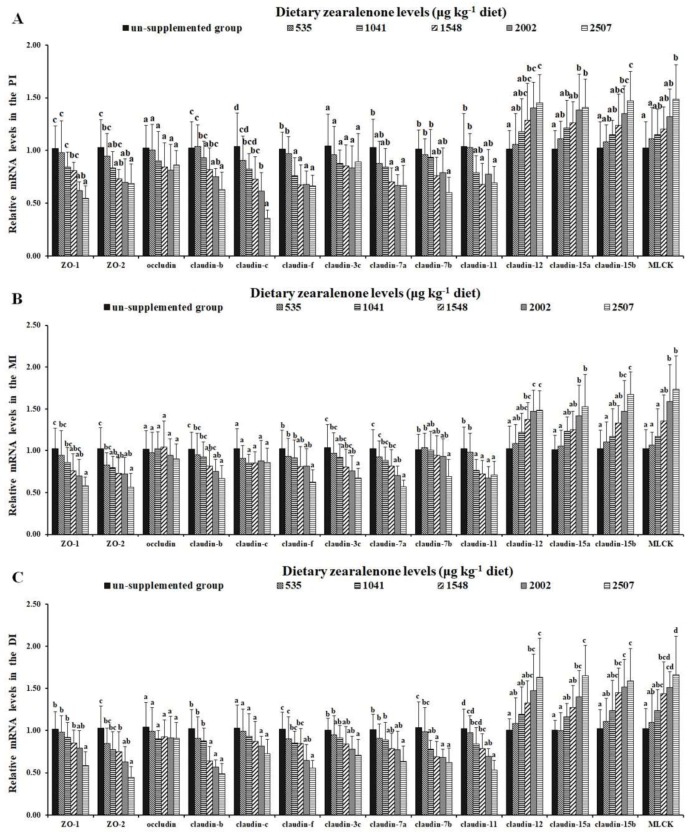
Relative mRNA levels of *zonula occludens-1 (ZO-1), ZO-2, occludin, claudin-b, claudin-c, claudin-f, claudin-3c, claudin-7a, claudin-7b, claudin-11, claudin-12, claudin-15a, claudin-15b*, and *MLCK* in the PI (**A**), MI (**B**), and DI (**C**) of juvenile grass carp fed diets containing graded levels of zearalenone. Data represent means of six fish in each group, error bars indicate SD. Values having different letters are significantly different (*p* < 0.05).

Figure 8 presents the influences of ZEA on the gene expressions of TJs in the intestines of fish. As shown in Figure 8A, the gene expression of *claudin-f* was markedly decreased in the 1041 μg/kg diet group (*p* < 0.05); mRNA levels of *zonula occludens-2* (*ZO-2), claudin-7a,* and *claudin-c* were markedly decreased in the 1548 μg/kg diet group (*p* < 0.05); mRNA levels of *ZO-1 and claudin-b* were markedly decreased in the 2002 μg/kg diet group (*p* < 0.05); and mRNA levels of *claudin-7b* and *claudin-11* were markedly reduced in the 2507 μg ZEA/kg diet group (*p* < 0.05) in the PI. As shown in Figure 8B, the gene expressions of *ZO-2* and *claudin-11* were markedly decreased in the 1041 μg/kg diet group (*p* < 0.05); mRNA levels of *ZO-1, claudin-b,* and clauidn*-7a* were markedly decreased in the 2002 μg/kg diet group (*p* < 0.05); and mRNA levels of *claudin-f* and *claudin-7b* were markedly reduced in the 2507 μg ZEA/kg diet group (*p* < 0.05) in the MI. As shown in Figure 8C, the gene expressions of *claudin-7b* and *claudin-f* were markedly decreased in the 1041 and 2002 μg/kg diet groups (*p* < 0.05), respectively; mRNA levels of *ZO-2, claudin-b,* and *claudin-11* were markedly decreased in the 1548 μg/kg diet group (*p* < 0.05); and mRNA levels of *ZO-1* and *claudin-7a* were markedly reduced in the 2507 μg ZEA/kg diet group (*p* < 0.05) in the DI. The mRNA levels of *claudin-3c* in the MI and DI were markedly decreased in the 2002 and 2507 μg ZEA/kg diet groups. The gene expressions of *claudin-12, claudin-15a,* and *claudin-15b* in the PI were markedly increased in the 2002 μg/kg diet group; mRNA levels of *MLCK* in the PI was markedly increased in the 2507 μg/kg diet group; mRNA levels of *claudin-15a, claudin-15b,* and *MLCK* in the MI were markedly increased in the 2002 μg/kg diet group; mRNA levels of *claudin-12* in the MI was markedly increased in the 1548 μg/kg diet group; the mRNA levels of *claudin-12* and *claudin-15a* in the DI were markedly increased in the 2002 μg/kg diet group; and the mRNA level of *claudin-15b* and *MLCK* in the DI were markedly increased in the 1548 μg/kg diet group. However, ZEA had no influence on the gene expressions of *occludin* in the PI, MI, or DI, on the gene expressions of *claudin-3c* in the PI, or on the gene expressions of *claudin-c* in the MI and DI (*p* > 0.05). 

### 2.6. Correlation Analyses

A correlation analysis of antioxidant capacity, apoptosis, and TJs-relevant indicator gene expressions in the intestines of fish is shown in Table S1 in the Supplementary Materials. The antioxidant-relevant indicators indicated that the activities of antioxidant enzymes showed a positive correlation with their homologous gene expressions (rather than *GSTP1* and *GSTP2* (not in DI)) in the intestines. These data confirm that the decline in the activities of antioxidant enzymes caused by ZEA may have been partly relevant to the down–regulation of their homologous gene levels in the fish intestines. The gene expressions (rather than *GSTP1* and *GSTP2* (not in DI)) of the antioxidant enzymes showed a positive correlation with nuclear Nrf2 protein expression in fish intestines. *Caspase-9* gene expression showed a positive correlation with gene expressions of pro-apoptotic *Apaf-1* as well as *Bax*, whereas it showed a negative correlation with gene expressions of anti-apoptotic *Bcl-2* as well as *Mcl-1* in fish intestines. Additionally, the gene expressions of *caspase-8* showed a positive correlation with the gene expressions of *FasL*, pro-apoptotic *Apaf-1,* and *Bax,* while the gene expression of *FasL* had a positive correlation with the gene expression of *JNK* in fish intestines. Furthermore, the gene expressions of barrier-forming TJs (rather than *claudin-c* in MI and DI, *claudin-3c* in PI) showed a negative correlation with the gene expression of *MLCK*, whereas the gene expressions of pore-forming TJs had a positive correlation with gene expression of *MLCK* in fish intestines.

## 3. Discussion

### 3.1. Growth Performance and Body Malformation of Fish

A previous study reported that toxicants can lead to growth retardation in fish [9]. In our study, diets with a ZEA content ≥ 1041 μg/kg depressed the levels of growth indicators (such as PWG, FI, and FE) and intestinal development indicators (IL, ILI, IW, and ISI) in fish. Based on broken-line analysis of PWG, the dietary dose of NOAEL for ZEA in fish was evaluated to be 496.22 μg/kg.

One of the reasons for the inhibition of grass carp growth is the deformity of the fish body, which could reflect the toxic effect of toxicants on fish [18,30]. Our study showed that diets with a ZEA content ≥ 1041 μg/kg caused body malformation, such as body line irregularities, upward curvature of the tail, caudal fin deformity, operculum abnormality, and skeletal anomalies in juvenile grass carp. A similar study reported that ZEA could cause upward curvature of the body axis in zebrafish [31]. The deformities observed in ZEA-treated grass carp in the present study were probably related to genotoxicity. One study reported that a dietary ZEA level of 621 μg/kg may be capable of causing genotoxicity in the common carp [32]. Additionally, genotoxicity leads to the occurrence of malformations in zebrafish [33]. 

Moreover, the growth of grass carp has previously been found to be correlated with intestinal structural integrity [34]. The destruction of intestinal structure might be related to histopathological damage [35]. Thus, we next studied the influence of ZEA on histopathological lesions in the grass carp intestine.

### 3.2. Intestinal Histopathological Lesions Caused by ZEA and Possible Mechanisms

Intestinal histopathological lesions can be caused by the accumulation of harmful substances [36]. The present study found that for dietary ZEA levels ≥ 535 μg/kg, ZEA accumulated in the intestines of fish. Meanwhile, it was observed that ZEA caused apparent symptoms of swelling and hyperemia in the fish intestines, and it is assumed that ZEA depressed the intestinal development of juvenile grass carp. Pathological intestinal changes in fish could be caused by toxicants [37]. After further histological analysis, we found that diets with a ZEA content ≥ 1041 μg/kg caused pathological changes, such as epithelial cell necrosis, edema in the lamina propria, and goblet cell hyperplasia, which might be the cause of the histopathological changes in the intestines of juvenile grass carp [7,35,38]. This suggests that ZEA could damage fish intestines. A similar study reported that ZEA could cause histological lesions (including necrotic areas, cytoplasm vacuolization, and macrophage aggregates) in the liver of rainbow trout [22]. 

Additionally, it has previously been found that the destruction of cellular structure and intercellular structure were partly responsible for histopathological lesions in animal tissues [27]. 

The production of excess amounts of ROS is considered to be an important factor leading to toxicity [39]. ROS can cause oxidative damage [40] and trigger apoptosis [41], leading to the destruction of cellular structure. We found that diets with a ZEA content ≥ 1041μg/kg enhanced the productions of ROS, caused MDA and PC, and weakened the antioxidant enzyme capacity of fish intestines. The laddering of DNA fragmentation is a widely used method to monitor cell apoptosis in *Meretrix meretrix* [42]. In our research, we found that ZEA could lead to DNA fragmentation in the fish intestine (Figure 6), suggesting that ZEA could aggravate apoptosis in the fish intestine. However, another report found that ZEA had no influence on the contents of MDA in the intestine of the common carp [24]. These differences may be due to the level of polyunsaturated fatty acid (PUFA) in intestines. PUFA is liable to cause oxidative injury [43]. Intestinal PUFA content was found to be higher in grass carp (57.1% of complete fatty acids) [44] than in common carp (42.1% of total fatty acids) [45]. Therefore, grass carp are more sensitive to ZEA-related oxidative damage than the common carp. 

The activity of antioxidant enzymes is closely related to mRNA levels [46]. CuZnSOD is mainly located in the cytoplasm of eukaryotic cells. It may catalyze the decomposition of the toxic substance O_2_^-^, which is produced during the oxidation process in organisms—which is H_2_O_2_ and oxygen—and could reduce the oxidative stress load of organisms. Nrf2 is an important transcription factor [47,48] which plays an important role in the regulation of CuZnSOD and other antioxidant enzyme gene expression. In the present study, analysis of the molecular effect of ZEA on antioxidation and apoptosis in fish intestines showed that diets with a ZEA level ≥ 1041 μg/kg decreased the antioxidant enzyme gene expressions of *CuZnSOD, MnSOD, CAT, GPx1a, GPx1b, GPx4a, GPx4b, GSTR, GSTO1, GSTO2,* and *GR* (except *GSTP1* and *GSTP2* (not in DI)) in the fish intestine, which was related to inhibition of the Nrf2 signaling pathway. Caspase is the key to programmed cell death. Caspase-3 is a common protease in apoptosis in mammals, which is essential for some processes related to cell division and apoptotic body formation [49]. The P38 mitogen–activated protein kinase (p38MAPK) signal molecule is a key factor regulating apoptosis [50,51]. In the present study, we observed that diets with a ZEA level ≥ 1548 μg/kg up-regulated the apoptosis promoters *caspase-2, caspase-8,* and *caspase-9*; and the apoptosis executors *caspase-3, caspase-7,* pro-apoptotic *Apaf-1, Bax,* and *FasL*; and also decreased the gene expressions of anti-apoptotic *Bcl-2* and *Mcl-1*, related to the up-regulation of *p38MAPK* and *JNK* signaling pathways in the fish intestine. These results suggest that ZEA decreased antioxidant capacity and aggravated apoptosis in the fish intestine, partially by the suppressing Nrf2 and the up-regulating of p38MAPK signaling pathways, respectively.

Regarding oxidative damage, we observed three interesting phenomena caused by ZEA in the grass carp intestine. Firstly, we observed that ZEA barely decreased the gene expression of *GSTP2* (not *GSTP1*) in the DI of fish. Secondly, we found that ZEA decreased the gene expression of *GSTP2* in the DI (but not the PI or MI) of juvenile fish, which may be partially explained by the content of ZEA in the intestine. As mentioned above, high levels of ZEA could decrease the gene expression of *GSTP2.* The present study showed that, in the 10th week of exposure, the residue of ZEA in DI was the highest among the three intestinal segments, which supports our assumption. Thirdly, ZEA merely increased the gene expressions of *Keap1a* (rather than *Keap1b*) in the fish intestine. 

Previous in vitro research has shown that ZEA impaired the cell membrane integrity of fish [52]. The structure and function of tight junction may be regulated by the MLCK signaling pathway [34]. Inflammatory cytokines or pathogenic bacteria may activate MLCK, which could enhance extracellular permeability [53,54]. Previous studies suggested that, in fish, the down-regulation of the mRNA levels of *ZO-1, ZO-2,* and *claudin-b* could damage physical barrier function in the intestine [55]. Regarding damage of the intercellular structure, our study firstly found that diets with a ZEA content ≥ 1041 μg/kg decreased the gene expressions of studied barrier-forming TJs (not the *occludin* in PI, MI, and DI; *claudin-3c* in PI; and *claudin-c* in MI and DI) and increased the gene expressions of pore-forming TJs (claudin-12 and -15) associated with the regulation of the *MLCK* signaling pathway in the fish intestine, suggesting that ZEA disrupted tight junctions. By chance, we observed three interesting phenomena in the TJs of the fish intestine that were caused by ZEA: first, ZEA showed no marked influence on the gene expression of *occludin* in the fish intestine; second, ZEA decreased the gene expressions of *claudin-3c* in the MI and DI (but not in the PI) of juvenile fish; and third, ZEA decreased the gene expressions of *claudin-c* in the PI (but not in the MI and DI) of juvenile fish.

### 3.3. The Dose of NOAEL for ZEA

As shown in Table 3, this study firstly revealed that, based on PWG, the dose of NOAEL for ZEA on juvenile grass carp (15.34–115.40g) was evaluated to be the 496.22 μg/kg diet. Meanwhile, on the basis of the productions of ROS in the DI, the doses of NOAEL for ZEA on juvenile grass carp were evaluated to be the 464.63 μg/kg diet. Based on the mRNA levels of caspase-8 and claudin-15b in DI, the dose of NOAEL for ZEA on juvenile grass carp were evaluated to be the 298.77 and 450.57 μg/kg diets, respectively. These data suggest that the stated values of ZEA which cause oxidative damage, apoptosis, and breached tight junctions are lower than the levels of ZEA that cause growth retardation, suggesting that lower levels of ZEA could affect the intestine in juvenile grass carp. Similarly, the stated values of ZEA on antioxidants are lower than the levels that caused growth retardation in postweaning gilts [56]. Our results indicate that the concentration of ZEA in the DI is the highest among the three intestinal segments of juvenile fish after 10 weeks of exposure, which agree with this evaluation results. Our finding is similar to that of Huang et al. [7], who found that the residue of DON in the DI of grass carp is the highest among the three intestinal segments. However, the dietary dose of NOAEL for ZEA for male rats is 1000 μg/kg [57], suggesting that juvenile grass carp are more sensitive to ZEA than rats.

## 4. Conclusions

**Figure 9:**
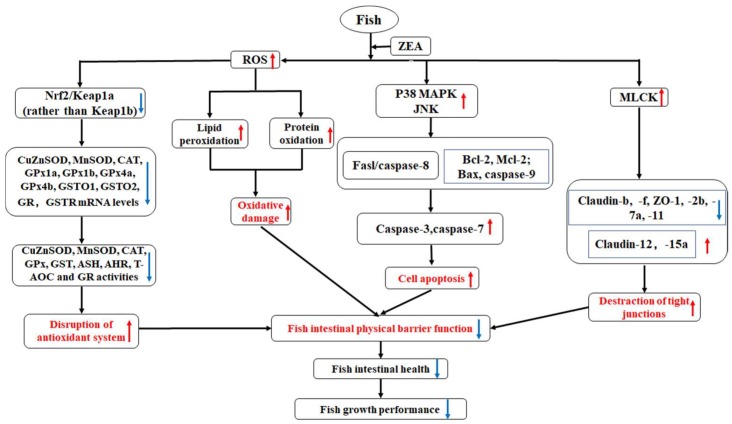
The potential action pathways of dietary ZEA-disrupted intestinal structural integrity in fish. Red arrows: up-regulation; blue arrows: down-regulation.

In conclusion, as presented in Figure 9, our study showed that ZEA decreased the growth performance and impaired the intestinal structural integrity of fish, as demonstrated by the following findings: (1) ZEA accumulated in the intestinal tissues and caused intestinal histopathological lesions in fish; (2) ZEA caused oxidative injury, aggravated cell apoptosis, and disrupted the tight junctions in fish intestines, which are probably related with the Nrf2, JNK, and MLCK signaling pathways, respectively. However, ZEA had no influence on the gene expressions of *Keap1b* (rather than *Keap1a*), *GSTP1,* or *GSTP2* (not in the DI), or on tight junctions *occludin*, *claudin-c* (not in the PI), and *claudin-3c* (not in the MI or DI). These inconspicuous phenomena require further study.

## 5. Materials and Methods 

### 5.1. Chemicals and Reagents

ZEA (purity > 98%) was purchased from Pribolab Pte, Ltd. (Tanjung Bago, Singapore). The RNAiso Plus kit was purchased from TaKaRa (Dalian, China). The Bio-Rad protein assay kit was purchased from Bio-Rad (Hercules, CA, USA). ECL Western Blotting Substrate Kit was purchased from Beyotime Biotechnology Inc. (Nantong, China). The ZEA ELISA kit was purchased from Clover Technology Group (Beijing, China). The enzyme-activity kit of antioxidant enzymes MDA, PC, ASA, AHR, SOD, CAT, GPx, GST, GR, and GSH were purchased from NanJing Jiancheng Bioengineering Institute (Nanjing, China). The ROS kit was purchased from Clover Technology Group (Beijing, China).

### 5.2. Diets

The elementary compositions of the diets are presented in Table 4. The main sources of dietary protein included fish meal, casein, and gelatin. Fish oil and soybean oil were the main sources of dietary lipid. Six different concentrations of ZEA were used, namely 0 (un-supplemented), 500, 1000, 1500, 2000, and 2500 μg/kg feed. The method of addition was according to Pietsch et al. [32]. Through high-performance liquid chromatography (HPLC), and following the method of Nuryono et al. [58], the dietary real ZEA concentrations were detected to be 0, 535, 1041, 1548, 2002, and 2507 μg/kg. The diets were preserved at −20 °C until feeding according to Pietsch et al. [32].

### 5.3. Experiments and Samples Collection 

For the experimental procedures, permission was obtained from the Animal Care and Use Committee of Sichuan Agricultural University, Chengdu, China, on 10 March 2017 (Approval No. 2017-0310). The grass carp were purchased from a fish farm in Sichuan, China, and adapted to the aquaculture circumstance for 4 weeks according to Ji et al. [62]. Afterwards, 1440 juvenile grass carp (mean weight 15.34 ± 0.06 g) were optionally allocated to 18 breeding cages (length, width, and height of 1.4 m), leading to 80 juvenile fish per cage. A tray was inserted in the bottom of each cage to gather the remaining feed on the basis of Sveier et al. [63]. Fish were offered several diets four times per day for 10 weeks. After feeding for half an hour, the remaining feed was collected, before being dried and weighed in other to calculate the FI according to Xu et al. [64]. In the trial period, the dissolved oxygen content of the aquatic environment was more than 6.0 mg/L, the water temperature was 28.0 ± 2.0 °C, and the water pH level was 7.4 ± 0.3. The growth experiment was carried out under natural light and dark cycles, analogous to our previous study [65].

At the end of the experiment, fish from each cage were weighted and counted. Then, 45 fish per treatment were randomly picked, before being hocused in a benzocaine bath according to Geraylou et al. [66]. After sacrifice, the intestines of the juvenile grass carp were segmented into PI, MI, and DI, then frozen in liquid nitrogen and preserved at −80 °C for subsequent analysis by the method of our former research [64]. 

### 5.4. Analysis of Feeding Components 

The components of the trial diets were analyzed based on the standard methods of Association of Official Analytical Chemists (AOAC) [67]. In short, we used the Kjeldahl nitrogen determination method to detect the amount of crude protein, and the Soxhlet extraction method to determine the amount of crude lipid. 

### 5.5. Histological Examination

Three fish intestines (rinsed with 0.9% saline) from each treatment were freely collected and stored in 4% paraformaldehyde for histological examination [68]. Preserved samples were sectioned (4 μm). The sections of intestine sample were stained with hematoxylin and eosin (H & E) and observed with a digital camera attached to a Nikon TS100 light microscope.

### 5.6. Biochemical Analysis

After the feeding trial, intestinal tissue was homogenized in 10 volumes (*w*/*v*) of ice-cold physiological saline and centrifuged at 6000× *g* for 20 min at a temperature of 4 °C [69]. Then, the supernatants were used to detect related indicators. Wang et al. assayed the ROS production [70]. The production of MDA was detected on the basis of Esterbauer and Cheeseman [71]. Deng et al. measured the production of GSH, and the activity of SOD, GR, and CAT [72]. GPx was analyzed as described by Flohé and Günzler [73]. The content of PC was determined referring to Baltacıoğlu et al. [74]. The activities of ASA, AHR, and GST were analyzed by the method of Jiang et al. [75]. The contents of ZEA in fish intestinal tissue were measured by ELISA referring to Liu et al. [27]. The contents of ZEA are presented in Table 1.

### 5.7. DNA Fragmentation

The DNA fragmentation of the intestinal samples was measured according to Jiang et al. [76]. The fragmented DNA was detected through electrophoresis using 2% agarose gel. The agarose gel was photographed using the Gene Genius Bio-Imaging system (ChemiDoc^TM^ XRS+, Syngene, Frederick, MD, USA).

### 5.8. RT-PCR Analysis

RNA was extracted from the three intestinal segments using an RNAiso Plus kit. Agarose gel (1%) electrophoresis and NanoDrop 2000 (Thermo Fisher Scientific, Wilmington, DE, USA) analysis were used to evaluate the quality and quantity of RNA, respectively. Afterwards, complementary DNA was synthesized using the PrimeScript RT reagent Kit (TaKaRa). The primers for RT-PCR are presented in Table. Table S2 of the Supplementary Materials, which were referred to the sequences in our former research, as well as the published sequences in grass carp. Based on the results of our elementary estimate of reference genes (data not shown), *β-actin* was chosen as a reference gene to normalize the results. The 2^−ΔΔCT^ method was applied to compute the gene expression data after demonstrating that the primers were enlarged with an efficiency of about 100% as represented, according to Livak and Schmittgen [77].

### 5.9. Western Blot

The protein homogenate of fish intestine and antibodies, as well as western blotting, were performed as in our previous study [76]. The protein concentration was measured using the Bio-Rad protein assay kit. Protein samples were analyzed by SDS-PAGE and shifted to a polyvinylidene fluoride (PVDF) membrane. After that, the membrane was closed for 1 h at room temperature and hatched with the antibodies Nrf2 and Lamin B1 overnight at 4 °C [78]. Lamin B1 was used as a reference protein for nuclear Nrf2. After cleaning, the PVDF membrane was hatched for 2 h with a secondary antibody. The membranes were observed by ECL reagents. The western blots were analyzed using National Institutes of Health (NIH) Image 1.63 software. We performed this trial in triplicate, acquiring similar results each time.

### 5.10. Statistical Analysis

The trial data were displayed as mean ± standard deviation. The significant differences among treatments was evaluated by the one-way analysis of variance (ANOVA) with Duncan’s multiple-range test at *p* < 0.05 using the SPSS 18.0 software (SPSS Inc., Chicago, IL, USA). The IBW, FBW, FI, PWG, SGR, and FE were calculated according to Tang et al. [79]. ILI and ISI were calculate by the method of Jiang et al. [80]. As presented in Table 5, on the basis of the PWG, the production of ROS, the mRNA levels of caspase-8 and claudin-15b in the DI, and the doses of NOAEL for ZEA in juvenile grass carp were determined referring to Wang et al. [81]. 

## Figures and Tables

**Table 1:** Growth performance, intestinal growth, and zearalenone (ZEA) concentration (μg/kg tissue) in the proximal intestine (PI), mid intestine (MI), and distal intestine (DI) of juvenile grass carp (*Ctenopharyngodon idellus*) fed the diets with graded levels of ZEA for 10 weeks.

Growth Indicators	Dietary ZEA Levels (μg/kg Diet)					
	0	535	1041	1548	2002	2507
**IBW ^1^**	15.35 ± 0.07 ^a^	15.31 ± 0.03 ^a^	15.29 ± 0.06 ^a^	15.36 ± 0.15 ^a^	15.29 ± 0.04 ^a^	15.45 ± 0.05 ^a^
**FBW ^1^**	115.40 ± 1.11 ^e^	114.73 ± 0.68 ^e^	105.83 ± 1.81 ^d^	92.21 ± 0.97 ^c^	85.80 ± 1.12 ^b^	78.83 ± 0.39 ^a^
**PWG ^1^**	651.99 ±9.73 ^e^	649.20 ± 5.79 ^e^	592.01 ± 5.27 ^d^	500.31 ± 1.86 ^c^	461.04 ± 8.37 ^b^	410.32 ± 1.00 ^a^
**SGR ^1^**	2.882 ± 0.02 ^e^	2.877 ± 0.01 ^e^	2.763 ± 0.01 ^d^	2.560 ± 0.004 ^c^	2.464 ± 0.02 ^b^	2.328 ± 0.003 ^a^
**FI ^1^**	118.47 ± 0.84 ^e^	118.34 ± 0.91 ^e^	112.11 ± 0.11 ^d^	102.73 ± 0.79 ^c^	95.68 ± 0.73 ^b^	90.02 ± 0.68 ^a^
**FE ^1^**	0.845 ± 0.06 ^d^	0.840 ± 0.05 ^d^	0.808 ± 0.04 ^c^	0.748 ± 0.02 ^b^	0.737 ± 0.02 ^b^	0.704 ± 0.01 ^a^
**IL ^2^**	31.85 ± 1.61 ^d^	30.72 ± 0.44 ^d^	28.49 ± 0.41 ^c^	25.47 ± 0.50 ^b^	24.27 ± 0.24 ^ab^	23.95 ± 0.10 ^a^
**ILI ^2^**	140.89 ± 8.85 ^c^	139.75 ± 2.93 ^c^	131.41 ± 1.35 ^b^	121.18 ± 1.53 ^a^	120.08 ± 1.63 ^a^	118.55 ± 1.92 ^a^
**IW ^2^**	2.39 ± 0.12 ^d^	2.24 ± 0.12 ^d^	1.97 ± 0.05 ^c^	1.64 ± 0.12 ^b^	1.47 ± 0.04 ^ab^	1.44 ± 0.11 ^a^
**ISI ^2^**	1.99 ± 0.09 ^c^	2.00 ± 0.07 ^c^	1.83 ± 0.03 ^b^	1.71 ± 0.07 ^a^	1.69 ± 0.03 ^a^	2.49 ± 0.07 ^a^
**ZEA Content (ug/kg tissue)**						
**PI ^3^**	n.d ^4^	29.64 ± 2.62 ^a^	41.26 ± 4.92 ^b^	56.82 ± 5.88 ^c^	69.19 ± 6.39 ^d^	87.97 ± 6.94 ^e^
**MI ^3^**	n.d ^4^	34.44 ± 3.15 ^a^	49.61 ± 4.89 ^b^	72.39 ± 5.80 ^c^	81.62 ± 7.91 ^c^	95.56 ± 12.78 ^d^
**DI ^3^**	n.d ^4^	37.02 ± 3.67 ^a^	58.09 ± 3.71 ^b^	76.34 ± 5.64 ^c^	87.48 ± 6.95 ^d^	102.46 ± 9.95 ^e^

^1^ Values are means ± SD for three replicate groups, with 80 fish in each group, and different superscripts in the same row are significantly different (*p* < 0.05). IBW, initial body weight (g/fish); FBW, final body weight (g/fish); PWG, percentage of weight gain (%); SGR, specific growth rate; FI, feed intake (g/fish); FE, feed efficiency. ^2^ Values are means ± SD (*n* = 45), and different superscripts in the same row are significantly different (*p* < 0.05). IL, intestine length (cm); ILI, intestine length index; IW, intestine weight (g/fish); ISI, intestinal somatic index. ^3^ Values are means ± SD (*n* = 6), and different superscripts in the same row are significantly different (*p* < 0.05). ^4^ n.d: not detected.

**Table 2:** Oxidative damage and antioxidant capacity related parameters in the PI, MI, and DI of juvenile grass carp (*Ctenopharyngodon idella*) fed diets containing graded levels of ZEA for 10 weeks.

Item	Dietary ZEA Level (μg/kg Diet)					
	0	535	1041	1548	2002	2507
**PI**						
ROS	100.00 ± 10.97 ^a^	109.66 ± 8.81 ^ab^	113.90 ± 11.02 ^ab^	119.85 ± 12.10 ^bc^	131.45 ± 14.37 ^cd^	140.61 ± 12.95 ^d^
MDA	28.28 ± 1.72 ^a^	28.29 ± 2.74 ^a^	32.46 ± 3.16 ^b^	32.43 ± 2.53 ^b^	34.16 ± 3.45 ^b^	41.85 ± 3.43 ^c^
PC	5.82 ± 0.47 ^a^	5.88 ± 0.56 ^a^	6.90 ± 0.67 ^b^	7.56 ± 0.78 ^bc^	8.34 ± 0.87 ^cd^	8.59 ± 0.84 ^d^
ASA	184.37 ± 18.31 ^b^	186.21 ± 6.73 ^b^	181.99 ± 7.02 ^ab^	173.27 ± 12.90 ^ab^	171.67 ± 11.56 ^ab^	168.92 ± 6.46 ^a^
AHR	70.97 ± 6.67 ^b^	70.17 ± 4.72 ^b^	66.51 ± 3.01 ^ab^	65.73 ± 5.52 ^ab^	64.91 ± 4.56 ^ab^	62.05 ± 4.12 ^a^
CuZnSOD	11.17 ± 1.06 ^b^	10.42 ± 0.90 ^ab^	9.95 ± 0.80 ^a^	9.76 ± 0.28 ^a^	9.79 ± 0.84 ^a^	9.44 ± 0.32 ^a^
MnSOD	6.53 ± 0.45 ^b^	6.54 ± 0.39 ^b^	6.32 ± 0.61 ^ab^	6.67 ± 0.67 ^b^	5.83 ± 0.54 ^a^	5.67 ± 0.60 ^a^
CAT	3.37 ± 0.29 ^c^	3.31 ± 0.33 ^c^	3.07 ± 0.26 ^bc^	2.81 ± 0.21 ^b^	2.16 ± 0.16 ^a^	2.14 ± 0.22 ^a^
GPx	145.54 ± 12.87 ^c^	141.71 ± 13.58 ^bc^	145.81 ± 11.30 ^c^	132.24 ± 17.23 ^abc^	124.16 ± 10.42 ^ab^	126.55 ± 12.54 ^a^
GST	61.88 ± 5.65 ^e^	62.94 ± 4.90 ^e^	54.02 ± 5.57 ^d^	46.43 ± 3.73 ^c^	38.41 ± 3.56 ^b^	32.98 ± 3.19 ^a^
GR	54.16 ± 4.48 ^e^	52.37 ± 4.63 ^e^	41.47 ± 3.96 ^d^	35.07 ± 2.98 ^c^	28.57 ± 3.57 ^b^	24.09 ± 2.13 ^a^
GSH	16.11 ± 1.27 ^b^	15.94 ± 1.17 ^b^	15.44 ± 1.65 ^ab^	14.58 ± 1.33 ^ab^	14.48 ± 1.42 ^ab^	14.05 ± 1.13 ^a^
**MI**						
ROS	100.00 ± 9.38 ^a^	104.97 ± 3.32 ^ab^	116.17 ± 13.25 ^bc^	118.34 ± 7.52 ^c^	137.31 ± 11.22 ^d^	136.91 ± 9.74 ^d^
MDA	27.60 ± 2.43 ^a^	27.94 ± 2.85 ^a^	30.43 ± 3.26 ^ab^	33.32 ± 2.99 ^bc^	33.37 ± 1.83 ^bc^	34.67 ± 3.34 ^c^
PC	4.76 ± 0.42 ^a^	5.13 ± 0.49 ^a^	5.90 ± 0.54 ^b^	6.30 ± 0.57 ^b^	7.18 ± 0.34 ^c^	7.84 ± 0.92 ^c^
ASA	204.90 ± 19.85 ^c^	197.82 ± 13.47 ^bc^	182.98 ± 15.94 ^b^	161.31 ± 14.56 ^a^	159.77 ± 5.22 ^a^	154.46 ± 11.51 ^a^
AHR	69.87 ± 6.96 ^b^	68.91 ± 4.40 ^b^	65.31 ± 6.09 ^ab^	63.94 ± 3.41 ^ab^	60.08 ± 5.77 ^a^	58.77 ± 4.48 ^a^
CuZnSOD	9.26 ± 0.81 ^c^	9.05 ± 0.80 ^bc^	8.49 ± 0.26 ^ab^	8.34 ± 0.42 ^a^	8.24 ± 0.30 ^a^	8.21 ± 0.40 ^a^
MnSOD	6.55 ± 0.64 ^b^	6.48 ± 0.72 ^b^	6.02 ± 0.52 ^ab^	5.41 ± 0.47 ^a^	5.41 ± 0.35 ^a^	5.36 ± 0.24 ^a^
CAT	3.05 ± 0.30 ^b^	3.03 ± 0.29 ^b^	2.83 ± 0.24 ^ab^	2.75 ± 0.23 ^ab^	2.74 ± 0.24 ^ab^	2.55 ± 0.22 ^a^
GPx	130.65 ± 12.54 ^c^	127.53 ± 12.08 ^bc^	119.01 ± 10.78 ^abc^	114.97 ± 6.79 ^ab^	117.43 ± 4.18 ^ab^	110.51 ± 11.50 ^a^
GST	56.54 ± 5.20 ^c^	55.32 ± 5.54 ^c^	48.17 ± 4.24 ^b^	47.96 ± 3.33 ^b^	42.76 ± 4.37 ^a^	31.95 ± 2.69 ^a^
GR	35.53 ± 2.61 ^e^	29.00 ± 2.35 ^d^	28.53 ± 2.06 ^d^	25.34 ± 2.26 ^c^	21.43 ± 1.60 ^b^	17.77 ± 1.47 ^a^
GSH	15.39 ± 1.51 ^b^	15.26 ± 1.11 ^ab^	14.78 ± 0.41 ^ab^	14.26 ± 1.50 ^ab^	13.90 ± 1.20 ^ab^	13.74 ± 1.05 ^a^
**DI**						
ROS	100.00 ± 8.85 ^a^	104.89 ± 8.97 ^ab^	115.86 ± 11.41 ^bc^	120.04 ± 10.65 ^c^	140.72 ± 13.19 ^d^	145.75 ± 9.05 ^d^
MDA	31.61 ± 2.85 ^a^	34.02 ± 2.83 ^ab^	34.04 ± 3.31 ^ab^	36.02 ± 2.40 ^b^	36.69 ± 2.96 ^b^	42.71 ± 4.11 ^c^
PC	4.96 ± 0.43 ^a^	5.46 ± 0.42 ^ab^	6.38 ± 0.55 ^bc^	6.06 ± 0.59 ^c^	6.46 ± 0.69 ^c^	7.42 ± 0.68 ^d^
ASA	170.19 ± 14.86^b^	166.70 ± 9.58 ^b^	162.05 ± 7.84 ^b^	147.57 ± 13.79 ^a^	143.45 ± 10.63 ^a^	134.84 ± 3.42 ^a^
AHR	51.85 ± 2.66 ^d^	50.86 ± 3.55 ^d^	47.95 ± 3.49 ^cd^	45.80 ± 2.34 ^bc^	42.18 ± 4.12 ^b^	37.07 ± 3.21 ^a^
CuZnSOD	8.18 ± 0.61 ^b^	8.18 ± 0.35 ^b^	8.14 ± 0.44 ^b^	7.36 ± 0.61 ^a^	7.25 ± 0.58 ^a^	7.04 ± 0.46 ^a^
MnSOD	5.98 ± 0.42 ^c^	6.00 ± 0.24 ^c^	5.56 ± 0.30 ^bc^	5.13 ± 0.48 ^ab^	5.07 ± 0.46 ^ab^	5.03 ± 0.44 ^a^
CAT	2.76 ± 0.22 ^c^	2.64 ± 0.26 ^c^	2.55 ± 0.22 ^c^	2.29 ± 0.20 ^b^	2.21 ± 0.19 ^b^	1.97 ± 0.13 ^a^
GPx	94.06 ± 9.11 ^d^	86.38 ± 8.29 ^cd^	78.56 ± 6.89 ^bc^	74.85 ± 7.34 ^b^	62.34 ± 5.68 ^a^	56.72 ± 5.59 ^a^
GST	48.14 ± 4.47 ^d^	46.82 ± 4.36 ^d^	41.32 ± 3.58 ^c^	38.57 ± 3.13 c	31.30 ± 3.68 ^b^	25.41 ± 2.56 ^a^
GR	46.44 ± 4.61 ^d^	45.04 ± 2.66 ^d^	37.83 ± 3.76 ^c^	34.93 ± 3.43 ^bc^	32.43 ± 2.79 ^b^	27.59 ± 2.48 ^a^
GSH	13.43 ± 1.25 ^b^	13.34 ± 1.06 ^b^	12.75 ± 1.18 ^ab^	12.56 ± 1.18 ^ab^	12.35 ± 1.15 ^ab^	11.56 ± 0.81 ^a^

Values are means ± SD (*n* = 6), and superscripted different letters in the same row are significantly different (*p* < 0.05). ROS, reactive oxygen species (% dichlorofluorescein florescence); MDA, malondialdehyde (nmol/g tissue); PC, protein carbonyl (nmol/mg protein); ASA, anti-superoxide anion (U/g protein); AHR, anti-hydroxyl radical (U/mg protein); CuZnSOD, copper/zinc superoxide dismutase (U/mg protein); MnSOD, manganese superoxide dismutase (U/mg protein); CAT, catalase (U/mg protein); GPx, glutathione peroxidase (U/mg protein); GST, glutathione-S-transferase (U/mg protein); GR, glutathione reductase (U/g protein); GSH, glutathione (mg/g protein).

**Table 3:** The doses of no observed adverse effect levels (NOAEL) for ZEA that were evaluated for broken-line analysis on different indexes (μg/kg).

Indicators	Broken-Line Analysis	*R* ^2^	The Doses of NOAEL for ZEA (μg/kg)
PWG	Y = −0.1243x + 712.2785; Y_max_ = 650.60	0.9847	496.22
ROS	Y = 0.0216x + 92.408; Y_min_ = 102.44	0.9481	464.63
Caspase-8	Y = 0.0002x + 1.2174; Y_min_ = 1.16	0.9778	298.77
Claudin-15b	Y = 0.0002x + 1.0318; Y_min_ = 1.12	0.9203	450.57

**Table 4:** Composition and nutrients content of basal diet.

Ingredients	Content (%)	Nutrients	Content (%)
Fish meal	5.50	Crude protein ^4^	32.24
Casein	21.67	Crude lipid ^4^	5.03
Gelatin	6.00	n-3 ^5^	1.04
α-starch	23.00	n-6 ^5^	0.96
Rice flour	25.74	Available phosphorus^6^	0.84
Fish oil	2.52		
Soybean oil	1.81		
Cellulose	5.00		
Ca(H_2_PO4)_2_	3.16		
Vitamin premix ^1^	1.00		
Mineral premix ^2^	2.00		
Choline chloride (50%)	1.00		
ZEA premix ^3^	1.00		
Ethoxyquin (30%)	0.05		
DL-Methionine (99%)	0.45		
L-Tryptophan (99.2%)	0.05		
Threonine (98.5%)	0.05		

^1^ Per kilogram of vitamin premix (g/kg): retinyl acetate (500,000 IU/g), 0.8g; cholecalciferol (500,000 IU/g), 0.32g; D, L-α-tocopherol acetate (50%), 40g; menadione (22.9%), 0.83g; cyanocobalamin (1%), 0.94g; D-biotin (2%), 0.75g; folic acid (95%), 0.379g; thiamine nitrate (98%), 0.144g; ascorhyl acetate (95%), 4.737g; niacin (99%), 2.576g; meso-inositol (98%), 21.84g; calcium-D-pantothenate (98%), 2.772g; riboflavin (80%), 0.775g; pyridoxine hydrochloride (98%), 0.619g. All ingredients were diluted with corn starch to 1 kg. ^2^ Per kilogram of mineral premix (g/kg): MnSO_4_⋅H_2_O (31.8% Mn), 3.098g; MgSO_4_⋅H_2_O (15.0% Mg), 237.84g; FeSO_4_⋅H_2_O (30.0% Fe), 15g; ZnSO_4_⋅H_2_O (34.5% Zn), 7.68g; CuSO_4_⋅5H_2_O (25.0% Cu), 0.6g; KI (76.9% I), 0.067g; Na_2_SeO3 (44.7% Se), 0.132g. All ingredients were diluted with corn starch to 1 kg. ^3^ Per kilogram of ZEA premix (g/kg): the ZEA was diluted with corn starch to 1 kg. Premix was added to obtain graded levels of ZEA. ^4^ Crude protein and crude lipid contents were measured value. ^5^ n-3 and n-6 contents were in reference to Zeng et al. [59], and calculated according to NRC (2011) [60]. ^6^ Available phosphorus content was in reference to Liang et al [61], and calculated according to NRC (2011) [60].

**Table 5:** Computational formula.

Growth Indicators	Formula
PWG	100 × [FBW (g/fish) − IBW (g/fish)]/IBW (g/fish)
SGR	100 × [In (mean final weight − In (mean initial weight)]/days
FE	[FBW (g/fish) − IBW (g/fish)]/FI (g/fish)
ILI	100 × intestine length (cm)/total body length (cm)
ISI	100 × wet intestine weight (g)/wet body weight (g)

## Supplementary Materials

The following are available online at https://www.mdpi.com/2072-6651/11/6/333/s1, Table S1: Correlation coefficient of parameters in the PI, MI, and DI of juvenile grass carp. Table S2: Real-time PCR primer sequences.
