# Supplementary material for: Effects of Dietary Zearalenone on Oxidative Stress, Cell Apoptosis, and Tight Junction in the Intestine of Juvenile Grass Carp (Ctenopharyngodon idella)
Source: Toxins (Basel). 2019 Jun 12;11(6):333. doi: 10.3390/toxins11060333 (PMC6628422; doi:10.3390/toxins11060333)
Supplement: Supplementary file 1 [file toxins-11-00333-s001.pdf]

Supplementary Materials: Supplementary Materials: Effects of Dietary Zearalenone on Oxidative Stress, Cell Apoptosis, and Tight Junction in the Intestine of Juvenile Grass Carp (*Ctenopharyngodon idella*)

Ya-Li Wang, Xiao-Qiu Zhou, Wei-Dan Jiang, Pei Wu, Yang Liu, Jun Jiang, Shang-Wen Wang, Sheng-Yao Kuang, Ling Tang and Lin Feng

Table S1. Correlation coefficient of parameters in the PI, MI, and DI of juvenile grass carp.

Independent Parameters	Dependent Parameters	PI		MI		DI	
		Correlation Coefficients	*p*	Correlation Coefficients	*p*	Correlation Coefficients	*p*
CuZnSOD mRNA level	CuZnSOD activity	+0.957	<0.01	+0.941	<0.01	+0.749	=0.086
MnSOD mRNA level	MnSOD activity	+0.580	=0.227	+0.917	<0.05	+0.905	<0.05
CAT mRNA level	CAT activity	+0.987	<0.01	+0.902	<0.05	+0.964	<0.01
GPx1a mRNA level	GPx activity	+0.955	<0.01	+0.890	<0.05	+0.840	<0.05
GPx1b mRNA level		+0.745	=0.089	+0.928	<0.01	+0.885	<0.05
GPx4a mRNA level		+0.888	<0.05	+0.911	<0.05	+0.992	<0.01
GPx4b mRNA level		+0.927	<0.01	+0.934	<0.01	+0.962	<0.01
GSTR mRNA level	GST activity	+0.965	<0.01	+0.981	<0.01	+0. 985	<0.01
GSTO1 mRNA level		+0.865	<0.05	+0.870	<0.05	+0.993	<0.01
GSTO2 mRNA level		+0.742	=0.091	+0.876	<0.05	+0.827	<0.05
GR mRNA level	GR activity	+0.974	<0.01	+0.991	<0.01	+0.981	<0.01
Nuclear Nrf2 protein level	CuZnSOD mRNA level	+0.944	<0.01	+0.923	<0.01	+0.970	<0.01
	MnSOD mRNA level	+0.929	<0.01	+0.936	<0.01	+0.944	<0.01
	CAT mRNA level	+0.942	<0.01	+0.917	<0.01	+0.911	<0.05
	GPx1a mRNA level	+0.755	=0.082	+0.848	<0.05	+0.824	<0.05
	GPx1b mRNA level	+0.803	=0.054	+0.861	<0.05	+0.883	<0.05
	GPx4a mRNA level	+0.975	<0.01	+0.931	<0.01	+0.863	<0.05
	GPx4b mRNA level	+0.976	<0.01	+0.764	=0.077	+0.927	<0.01
	GSTR mRNA level	+0.963	<0.01	+0.911	<0.05	+0.990	<0.01
	GSTO1 mRNA level	+0.883	<0.05	+0.821	<0.05	+0.869	<0.05
	GSTO2 mRNA level	+0.876	=0.05	+0.704	=0.119	+0.722	<0.05

	GR mRNA level	+0.944	<0.01	+0.926	<0.01	+0.918	<0.05
	Keap1a mRNA level	−0.976	<0.01	−0.930	<0.05	−0.988	<0.01
Caspase-9 mRNA level	Apaf-1 mRNA level	+0.947	<0.01	+0.990	<0.01	+0.961	<0.01
	Bax mRNA level	+0.976	<0.01	+0.954	<0.01	+0.975	<0.01
	Bcl-2 mRNA level	−0.977	<0.01	−0.965	<0.01	−0.973	<0.01
	Mcl-1 mRNA level	−0.990	<0.01	−0.854	<0.05	−0.961	<0.01
JNK mRNA level	Apaf-1 mRNA level	+0.973	<0.01	+0.976	<0.01	+0.915	<0.05
	Bax mRNA level	+0.993	<0.01	+0.983	<0.01	+0.947	<0.01
	Bcl-2 mRNA level	−0.986	<0.01	−0.880	<0.05	−0.964	<0.01
	Mcl-1 mRNA level	−0.993	<0.01	−0.964	<0.01	−0.968	<0.01
Fasl mRNA level	Caspase-8 mRNA level	+0.970	<0.01	+0.983	<0.01	+0.960	<0.01
P38 MAPK mRNA level	FasL mRNA level	+0.922	<0.01	+0.987	<0.01	+0.909	<0.05
MLCK mRNA level	ZO-1 mRNA level	−0.968	<0.01	−0.979	<0.01	−0.951	<0.01
	ZO-2 mRNA level	−0.878	<0.05	−0.884	<0.05	−0.951	<0.01
	Occludin mRNA level	−0.773	=0.071	+0.702	=0.120	+0.790	=0.061
	Claudin-b mRNA level	−0.962	<0.01	−0.986	<0.01	−0.988	<0.01
	Claudin-c mRNA level	−0.997	<0.01	−0.575	=0.232	−0.992	<0.01
	Claudin-f mRNA level	−0.811	=0.05	−0.928	<0.01	−0.938	<0.01
	Claudin-3c mRNA level	−0.654	=0.158	−0.984	<0.01	−0.992	<0.01
	Claudin-7a mRNA level	−0.872	<0.05	−0.982	<0.01	−0.975	<0.01
	Claudin-7b mRNA level	−0.951	<0.01	−0.874	<0.01	−0.961	<0.01
	Claudin-11 mRNA level	−0.767	=0.075	−0.827	<0.05	−0.980	<0.01
	Claudin-12 mRNA level	+0.955	<0.01	+0.962	<0.01	+0.990	<0.01
	Claudin-15a mRNA level	+0.949	<0.01	+0.976	<0.01	+0.972	<0.01
	Claudin-15b mRNA level	+0.991	<0.01	+0.991	<0.01	+0.995	<0.01

Table S2. Real-time PCR primer sequences [1].

Target Gene	Primer Sequence Forward (5′→3′)	Primer Sequence Reverse (5′→3′)	Temperature (°C)	Accession Number
CuZnSOD	CGCACTTCAACCCTTACA	ACTTTCCTCATTGCCTCC	61.5	GU901214
MnSOD	ACGACCCAAGTCTCCCTA	ACCCTGTGGTTCTCCTCC	60.4	GU218534
CAT	GAAGTTCTACACCGATGAGG	CCAGAAATCCCAAACCAT	58.7	FJ560431
GPx1a	GGGCTGGTTATTCTGGGC	AGGCGATGTCATTCCTGTTC	61.5	EU828796
GPx1b	TTTTGTCCTTGAAGTATGTCCGTC	GGGTCGTTCATAAAGGGCATT	60.3	KT757315
GPx4a	TACGCTGAGAGAGGTTTACACAT	CTTTTCCATTGGGTTGTTCC	60.4	KU255598
GPx4b	CTGGAGAAATACAGGGGTTACG	CTCCTGCTTTCCGAACTGGT	60.3	KU255599
GSTP1	ACAGTTGCCCAAGTTCCAG	CCTCACAGTCGTTTTTTCCA	59.3	KM112099
GSTP2	TGCCTTGAAGATTATGCTGG	GCTGGCTTTTATTTCACCCT	59.3	KP125490
GSTO1	GGTGCTCAATGCCAAGGGAA	CTCAAACGGGTCGGATGGAA	58.4	KT757314
GSTO2	CTGCTCCCATCAGACCCATTT	TCTCCCCTTTTCTTGCCCATA	61.4	KU245630
GR	GTGTCCAACTTCTCCTGTG	ACTCTGGGGTCCAAAACG	59.4	JX854448
Nrf2	CTGGACGAGGAGACTGGA	ATCTGTGGTAGGTGGAAC	62.5	KF733814
Keap1a	TTCCACGCCCTCCTCAA	TGTACCCTCCCGCTATG	63.0	KF811013
Keap1b	TCTGCTGTATGCGGTGGGC	CTCCTCCATTCATCTTTCTCG	57.9	KJ729125
caspase-3	GCTGTGCTTCATTTGTTTG	TCTGAGATGTTATGGCTGTC	55.9	JQ793789
caspase-7	GCCATTACAGGATTGTTTCACC	CCTTATCTGTGCCATTGCGT	57.1	KT625601
caspase-8	ATCTGGTTGAAATCCGTGAA	TCCATCTGATGCCCATACAC	59.0	KM016991
caspase-9	CTGTGGCGGAGGTGAGAA	GTGCTGGAGGACATGGGAAT	59.0	JQ793787
Apaf1	AAGTTCTGGAGCCTGGACAC	AACTCAAGACCCCACAGCAC	61.4	KM279717
Bax	CATCTATGAGCGGGTTCGTC	TTTATGGCTGGGGTCACACA	60.3	JQ793788.1
FasL	AGGAAATGCCCGCACAAATG	AACCGCTTTCATTGACCTGGAG	61.4	KT445873
Bcl-2	AGGAAAATGGAGGTTGGGAT	CTGAGCAAAAAAGGCGATG	60.3	JQ713862.1
Mcl-1	TGGAAAGTCTCGTGGTAAAGCA	ATCGCTGAAGATTTCTGTTGCC	58.4	KT757307
JNK	ACAGCGTAGATGTGGGTGATT	GCTCAAGGTTGTGGTCATACG	62.3	KT757312
ZO-1	CGGTGTCTTCGTAGTCGG	CAGTTGGTTTGGGTTTCAG	59.4	KJ000055
ZO-2b	TACAGCGGGACTCTAAAATGG	TCACACGGTCGTTCTCAAAG	60.3	KM112095
occludin	TATCTGTATCACTACTGCGTCG	CATTCACCCAATCCTCCA	59.4	KF193855
claudin-b	GAGGGAATCTGGATGAGC	ATGGCAATGATGGTGAGA	57.0	KF193860
claudin-c	GAGGGAATCTGGATGAGC	CTGTTATGAAAGCGGCAC	59.4	KF193859
claudin-f	GCTGGAGTTGCCTGTCTTATTC	ACCAATCTCCCTCTTTTGTGTC	57.1	KM112097

claudin-7a	ACTTACCAGGGACTGTGGATGT	CACTATCATCAAAGCACGGGT	59.3	KT625604
claudin-7b	CTAACTGTGGTGGTGATGAC	AACAATGCTACAAAGGGCTG	59.3	KT445866
caudin-11	TCTCAACTGCTCTGTATCACTGC	TTTCTGGTTCACTTCCGAGG	62.3	KT445867
claudin-12	CCCTGAAGTGCCCACAA	GCGTATGTCACGGGAGAA	55.4	KF998571
claudin-15a	TGCTTTATTTCTTGGCTTTC	CTCGTACAGGGTTGAGGTG	59.0	KF193857
claudin-15b	AGTGTTCTAAGATAGGAGGGGAG	AGCCCTTCTCCGATTTCAT	62.3	KT757304
MLCK	GAAGGTCAGGGCATCTCA	GGGTCGGGCTTATCTACT	53.0	KM279719
β-actin	GGCTGTGCTGTCCCTGTA	GGGCATAACCCTCGTAGAT	61.4	M25013

[1] CuZnSOD, copper, zinc superoxide dismutase; MnSOD, manganese superoxide dismutase; CAT, catalase; GPx, glutathione peroxidase; GST, glutathione-S-transferase; GR, glutathione reductase; Nrf2, Nuclear factor-erythroid 2-related factor 2; Keap1, Kelch-like-ECH-associated protein 1; caspase, cysteinyl aspartic acid-protease; Apaf-1, apoptotic protease activating factor-1; Bax, Bcl-2 associated X protein; FasL, fas ligand; Bcl-2, B-cell lymphoma protein-2; Mcl-1, myeloid cell leukemia-1; JNK, c-Jun N-terminal protein kinase; ZO, zonula occludens; MLCK, myosin light chain kinase.
